# Supplementary material for: Planar chiral metasurfaces with maximal and tunable chiroptical response driven by bound states in the continuum
Source: Nat Commun. 2022 Jul 15;13:4111. doi: 10.1038/s41467-022-31877-1 (PMC9287326; doi:10.1038/s41467-022-31877-1)
Supplement: Supplementary file 1 — Supplementary Information [file 41467_2022_31877_MOESM1_ESM.pdf]

Supplementary Information:

Planar chiral metasurfaces with maximal and tunable chiroptical response driven by bound states in the continuum

Tan Shi[1,†], Zi-Lan Deng[1,†,*], Guangzhou Geng[2,†], Xianzhi Zeng[1], Yixuan Zeng[3], Guangwei Hu[3], Adam Overvig[4], Junjie Li[2,*], Cheng-Wei Qiu[5], Andrea Alù[4], Yuri S. Kivshar[6], and Xiangping Li[1,*]

[1]Guangdong Provincial Key Laboratory of Optical Fiber Sensing and Communications, Institute of Photonics Technology, Jinan University, Guangzhou 510632, China

[2]Beijing National Laboratory for Condensed Matter Physics, Institute of Physics, Chinese Academy of Sciences, Beijing 100191, China

[3]School of Electrical and Electronic Engineering, Nanyang Technological University, Singapore 639798, Singapore

[4]Photonics Initiative, Advanced Science Research Center, City University of New York, New York, NY 10031, USA

[5]Department of Electrical and Computer Engineering, National University of Singapore, Kent Ridge 117583, Republic of Singapore

[6]Nonlinear Physics Center, Research School of Physics, Australian National University, Canberra, ACT 2601, Australia

[†]These authors contributed equally to this work.

[*]E-mail: zilandeng@jnu.edu.cn; jjli@iphy.ac.cn; xiangpingli@jnu.edu.cn

Supplementary Notes

I. Temporal coupled-mode theory for the extrinsic chiral q-BICs.

We apply a two-mode temporal coupled-mode theory (TCMT) to the metasurface studied in the main text[1,2]. The two modes are the two Bloch resonances with opposite in-plane wave vectors. Based on the conservation of momentum, an incident wave, which has an in-plane wavevector $\boldsymbol{k}_{\parallel}$ = (k_x, k_y) above the light line and below the diffraction limit, will only couple to the resonances and outgoing waves with same $\boldsymbol{k}_{\parallel}$. We can write down the dynamic equations of TCMT:

$$\frac{dA}{dt} = (-i\omega_0 - \gamma)\boldsymbol{A} + K^T\boldsymbol{a}, \tag{S1}$$

$$\boldsymbol{b} = S\boldsymbol{a} = C\boldsymbol{a} + D\boldsymbol{A}, \tag{S2}$$

$$C = \begin{pmatrix} 0 & r_s & 0 & t_s & 0 & 0 & 0 & 0 \\ r_s & 0 & t_s & 0 & 0 & 0 & 0 & 0 \\ 0 & t_s & 0 & r_s & 0 & 0 & 0 & 0 \\ t_s & 0 & r_s & 0 & 0 & 0 & 0 & 0 \\ 0 & 0 & 0 & 0 & 0 & r_p & 0 & t_p \\ 0 & 0 & 0 & 0 & r_p & 0 & t_p & 0 \\ 0 & 0 & 0 & 0 & 0 & t_p & 0 & r_p \\ 0 & 0 & 0 & 0 & t_p & 0 & r_p & 0 \end{pmatrix}, \tag{S3}$$

where $\boldsymbol{A} = (A_1, A_2)^T$ is the complex amplitudes of the resonances (at $\boldsymbol{k}_{\parallel}$ and -$\boldsymbol{k}_{\parallel}$, respectively), ω_0 is the resonance frequency, γ is the decay rate due to radiation. Due to the reciprocity, the two resonances of opposite directions have the same frequency and decay rate[3]. $\boldsymbol{a} = [a_{s1}, a_{s2}, a_{s3}, a_{s4}, a_{p1}, a_{p2}, a_{p3}, a_{p4}]^T$ and $\boldsymbol{b} = [b_{s1}, b_{s2}, b_{s3}, b_{s4}, b_{p1}, b_{p2}, b_{p3}, b_{p4}]^T$ are the amplitudes of incoming and outgoing waves (Fig. S1). C is the scattering matrix for the direct (without the resonance) transmission and reflection through the slab (the Fabry-Perot background). Energy conservation and reciprocity constrain C to be unitary, symmetric, and identical for opposite wavevector directions. The subscript s(p) represents the radiation channel as s-polarization (p-polarization). The coupling matrices D and K between the resonances and the in and out plane waves are[3]

$$D = K\sigma_x = \begin{pmatrix} 0 & d_{s1} \\ d_{s2} & 0 \\ 0 & d_{s3} \\ d_{s4} & 0 \\ 0 & d_{p1} \\ d_{p2} & 0 \\ 0 & d_{p3} \\ d_{p4} & 0 \end{pmatrix}, \tag{S4}$$

where σ_x is the Pauli matrix. According to the energy conservation and time-reversal symmetry[2,3], we obtain

$$D^{\dagger}D = 2\gamma\boldsymbol{I}, \tag{S5}$$

$$D = K\sigma_x, \tag{S6}$$

$$CD^* = -D\sigma_x. \tag{S7}$$

Generally, the full scattering matrix including the direct pathway and resonance pathway is given by

$$S = C + \frac{DK^T}{i(\omega_0-\omega)+\gamma} = \left[I - \frac{DD^\dagger}{i(\omega_0-\omega)+\gamma}\right]C. \tag{S8}$$

For the incident plane wave propagating along $+\boldsymbol{k}_{\|}$, the scattering process can be shown as

$$b^- = S^{+k}a^+, \tag{S9}$$

where

$$a^+ = \begin{pmatrix} a_{s1} \\ a_{s3} \\ a_{p1} \\ a_{p3} \end{pmatrix},\ b^- = \begin{pmatrix} b_{s2} \\ b_{s4} \\ b_{p2} \\ b_{p4} \end{pmatrix}, \tag{S10}$$

$$S^{+k} = \begin{pmatrix} r_{ss} & t_{ss} & r_{ps} & t_{ps} \\ t_{ss} & r_{ss} & t_{ps} & r_{ps} \\ r_{sp} & t_{sp} & r_{pp} & t_{pp} \\ t_{sp} & r_{sp} & t_{pp} & r_{pp} \end{pmatrix} \tag{S11}$$

$$= \begin{pmatrix} r_s - \frac{r_s|d_{s2}|^2+t_s d_{s2}d_{s4}^*}{j(\omega_0-\omega)+\gamma} & t_s - \frac{t_s|d_{s2}|^2+r_s d_{s2}d_{s4}^*}{j(\omega_0-\omega)+\gamma} & -\frac{r_p d_{s2}d_{p2}^*+t_p d_{s2}d_{p4}^*}{j(\omega_0-\omega)+\gamma} & -\frac{t_p d_{s2}d_{p2}^*+r_p d_{s2}d_{p4}^*}{j(\omega_0-\omega)+\gamma} \\ t_s - \frac{t_s|d_{s4}|^2+r_s d_{s4}d_{s2}^*}{j(\omega_0-\omega)+\gamma} & r_s - \frac{r_s|d_{s4}|^2+t_s d_{s4}d_{s2}^*}{j(\omega_0-\omega)+\gamma} & -\frac{r_p d_{s4}d_{p2}^*+t_p d_{s4}d_{p4}^*}{j(\omega_0-\omega)+\gamma} & -\frac{t_p d_{s4}d_{p2}^*+r_p d_{s4}d_{p4}^*}{j(\omega_0-\omega)+\gamma} \\ -\frac{r_s d_{p2}d_{s2}^*+t_s d_{p2}d_{s4}^*}{j(\omega_0-\omega)+\gamma} & -\frac{t_s d_{p2}d_{s2}^*+r_s d_{p2}d_{s4}^*}{j(\omega_0-\omega)+\gamma} & r_p - \frac{r_p|d_{p2}|^2+t_p d_{p2}d_{p4}^*}{j(\omega_0-\omega)+\gamma} & t_p - \frac{t_p|d_{p2}|^2+r_p d_{p2}d_{p4}^*}{j(\omega_0-\omega)+\gamma} \\ -\frac{r_s d_{p4}d_{s2}^*+t_s d_{p4}d_{s4}^*}{j(\omega_0-\omega)+\gamma} & -\frac{t_s d_{p4}d_{s2}^*+r_s d_{p4}d_{s4}^*}{j(\omega_0-\omega)+\gamma} & t_p - \frac{t_p|d_{p4}|^2+r_p d_{p4}d_{p2}^*}{j(\omega_0-\omega)+\gamma} & r_p - \frac{r_p|d_{p4}|^2+t_p d_{p4}d_{p2}^*}{j(\omega_0-\omega)+\gamma} \end{pmatrix}.$$

Now, in order to discuss the effects of the Fabry-Perot background and simplify the discussion, we ignore the substrate and assume that the structure has z-axis mirror symmetry and inversion symmetry. Then, we can get $d_{s2} = \alpha_z d_{s4} = d_s$ and $d_{p2} = \alpha_z d_{p4} = d_p$, where α_z is a parity number to correlate the upside and downside coupling coefficients. Considering that the incidence is left-handed circularly polarized in x-y plane and from the 1 channel, we have

$$a^+ = \begin{pmatrix} a_s^1 \\ 0 \\ a_p^1 \\ 0 \end{pmatrix} = \sqrt{I_0}\begin{pmatrix} \frac{\sqrt{2}}{2} \\ 0 \\ \frac{i}{\cos\theta}\frac{\sqrt{2}}{2} \\ 0 \end{pmatrix}, \tag{S12}$$

where θ is the angle between the incidence and the z-axis. The outgoing wave would be

$$b^- = S^{+k}a^+ = \sqrt{I_0}\frac{\sqrt{2}}{2}\begin{pmatrix} r_{ss} + ir_{ps}/\cos\theta \\ t_{ss} + it_{ps}/\cos\theta \\ r_{sp} + ir_{pp}/\cos\theta \\ t_{sp} + it_{pp}/\cos\theta \end{pmatrix}. \tag{S13}$$

So, the reflectance and transmittance are simply[4]

$$R = \frac{1}{2}\left[\left|r_{pp} - ir_{ps}/\cos\theta\right|^2 + \left|r_{sp} - ir_{ss}/\cos\theta\right|^2\right], \tag{S14}$$

$$T = 1 - R. \tag{S15}$$

When the eigenmode is right-handed circularly polarized in the x-y plane, we have $d_s = -id_p\cos\theta$, thus the reflection on resonance would be[4]

$$R = \frac{|r_p|^2 + |r_s|^2/\cos^2\theta}{2}, \tag{S16}$$

which is the same as the reflection without resonance. Thus, CD occurs in this case, yet due to the presence of the Fabry-Perot background, the maximum of CD does not necessarily coincide with the *C* point.

II. Temporal coupled-mode theory for the intrinsic chiral q-BICs.

For the normal incidence case, we can assume that the background scattering matrix corresponds to a reflective birefringent effective medium.

$$C = \begin{pmatrix} r & t \\ t & r \end{pmatrix}, \tag{S17}$$

where,

$$r = \frac{i\cos(\Theta)}{2}\begin{bmatrix} \cos^2(\theta) + e^{i\Psi}\sin^2(\theta) & \left(1 - e^{i\Psi}\right)\cos(\theta)\sin(\theta) \\ \left(1 - e^{i\Psi}\right)\cos(\theta)\sin(\theta) & e^{i\Psi}\cos^2(\theta) + \sin^2(\theta) \end{bmatrix}, \tag{S18}$$

$$t = \frac{\sin(\Theta)}{2}\begin{bmatrix} \cos^2(\theta) + e^{i\Psi/2}\sin^2(\theta) & \left(1 - e^{i\Psi/2}\right)\cos(\theta)\sin(\theta) \\ \left(1 - e^{i\Psi/2}\right)\cos(\theta)\sin(\theta) & e^{i\Psi/2}\cos^2(\theta) + \sin^2(\theta) \end{bmatrix}, \tag{S19}$$

are the Jones matrices for background reflection and transmission, θ and Ψ are the orientation angle and phase difference of the birefringent effective medium, Θ is a free parameter modulating the overall transmission and reflection amplitude. In our present work, the orientation of the birefringence axis of the DSS structure is along $\theta = 45^{\circ}$, therefore the reflection and transmission Jones matrices reduce to,

$$r = \frac{i\cos(\Theta)}{2}\begin{bmatrix} 1 + e^{i\Psi} & 1 - e^{i\Psi} \\ 1 - e^{i\Psi} & 1 + e^{i\Psi} \end{bmatrix}, \tag{S20}$$

$$t = \frac{\sin(\Theta)}{2}\begin{bmatrix} 1 + e^{i\Psi/2} & 1 - e^{i\Psi/2} \\ 1 - e^{i\Psi/2} & 1 + e^{i\Psi/2} \end{bmatrix}. \tag{S21}$$

Planar form means the q-BIC scatters up/down symmetrically or anti-symmetrically. Therefore, the coupling matrix satisfies,

$$D = \begin{bmatrix} d_{s1} \\ d_{p1} \\ d_{s2} \\ d_{p2} \end{bmatrix}, \; d_{s1} = \pm d_{s2}\,, d_{p1} = \pm d_{p2}. \tag{S22}$$

Energy conservation requires that,

$$|d_{s1}|^2 + |d_{p1}|^2 = \gamma. \tag{S23}$$

Therefore, we can write the coupling coefficients as follows:

$$d_{s1} = \sqrt{\gamma}\cos(\alpha)\, e^{i\Phi_1}, \tag{S24}$$

$$d_{p1} = \sqrt{\gamma}\sin(\alpha)\, e^{i\Phi_2}. \tag{S25}$$

There are three unknowns, one of these degrees of freedom is unconstrained by TCMT, and it will depend on the perturbation chosen. Conservation of energy constraint specifies 2 of the 3 unknowns as follows,

$$\left(1 + e^{i\Psi}\right)d_{s1}^{*} + \left(1 - e^{i\Psi}\right)d_{p1}^{*} = -2d_{s1}, \tag{S26}$$

$$\left(1 + e^{i\Psi}\right)d_{p1}^{*} + \left(1 - e^{i\Psi}\right)d_{s1}^{*} = -2d_{p1}. \tag{S27}$$

If the effective background medium only has an isotropic response ($\Psi = 0$),

$$D = \sqrt{\gamma}\begin{bmatrix} \cos(\alpha) \\ \pm\sin(\alpha) \\ \cos(\alpha) \\ \pm\sin(\alpha) \end{bmatrix}. \tag{S28}$$

The coupled polarization is only linear polarization oriented at α, and no chirality could be supported.

If the effective background medium has a full birefringence response ($\Phi_1 = \Phi, \Phi_2 = \Phi + \pi$),

$$D = \sqrt{\frac{\gamma}{2}}\begin{bmatrix} e^{i\Phi} \\ -e^{-i\Phi} \\ e^{i\Phi} \\ -e^{-i\Phi} \end{bmatrix}. \tag{S29}$$

The coupled polarizations are elliptical polarizations crossing a great circle through ±45° on the Poincare sphere.

At the resonance condition $\omega = \omega_0$, and chose the reflection and transmission Jones matrix can be written as,

$$r = \frac{i\cos(\Theta)}{2}\begin{bmatrix} e^{2i\Phi} & 1 \\ 1 & e^{-2i\Phi} \end{bmatrix}, \tag{S30}$$

$$t = \frac{\sin(\Theta)}{2}\begin{bmatrix} e^{2i\Phi} & -1 \\ -1 & e^{-2i\Phi} \end{bmatrix}. \tag{S31}$$

We can clearly get a chiral response when

$$\Phi = \frac{\pi}{4}, \mathrm{D}= \frac{e^{\frac{i\pi}{4}}}{\sqrt{2/\gamma}}\begin{bmatrix}1\\ i\\ 1\\ i\end{bmatrix}, \tag{S32}$$

$$r = \frac{1}{2}\begin{bmatrix}i & 1\\ 1 & -i\end{bmatrix},\ t = \frac{1}{2}\begin{bmatrix}i & -1\\ -1 & -i\end{bmatrix}. \tag{S33}$$

For left-handed $|+\rangle = \frac{1}{\sqrt{2}}\begin{bmatrix}1\\ i\end{bmatrix}$ and right-handed $|-\rangle = \frac{1}{\sqrt{2}}\begin{bmatrix}1\\ -i\end{bmatrix}$ circularly polarized incidence, the outgoing wave would be

$$E_{r+} = r|+\rangle = i|-\rangle,\ E_{r-} = r|-\rangle = 0, \tag{S34}$$

$$E_{t+} = t|+\rangle = 0,\ E_{t-} = t|-\rangle = i|+\rangle. \tag{S35}$$

There is perfect unitary circular conversion dichroism for both transmission and reflection sides. Therefore, it suggests that, the perfect planar chirality can be designed by tuning the local response to be birefringent using a geometry that has an inversion center, and then breaking inversion symmetry with a suitable perturbation to yield an intrinsic planar chiral q-BIC.

Supplementary Figures

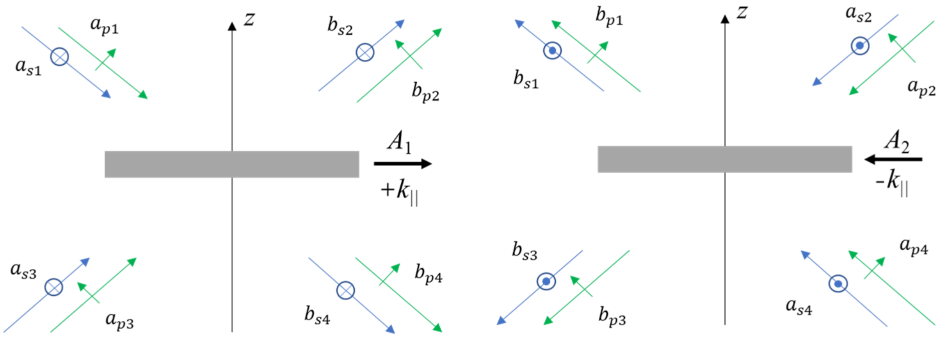

Figure S1. **Schematic illustration of the planar structure and the corresponding input and output channels in *s* and *p* polarizations.**

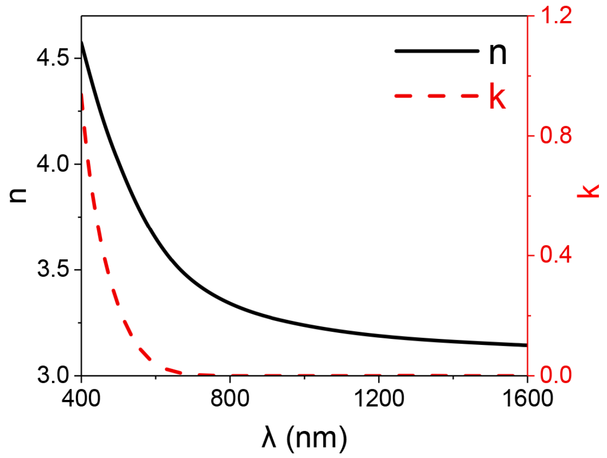

Figure S2. Experimentally measured optical parameters of α-Si. The refractive index (n) and extinction coefficient (k) are illustrated as black solid line and red dashed line, respectively.

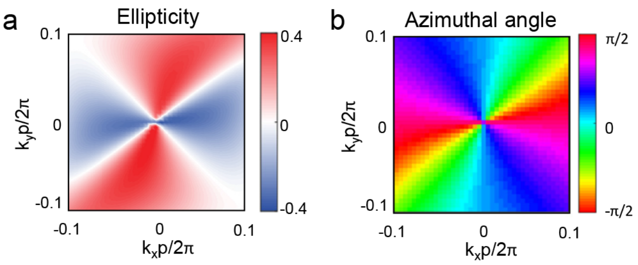

Figure S3. Color maps of the eigen-polarization states of the planar chiral q-BIC metasurface in the *k*-space. Polarization parameter (a: ellipticity $\chi=S_3/S_0$, b: azimuthal angle $\psi=Arg(S_1+iS_2)/2$, where, S_0, S_1, S_2, S_3 are Stokes parameters) maps in the *k*-space for the eigen-modes supported by the planar chiral q-BIC metasurface.

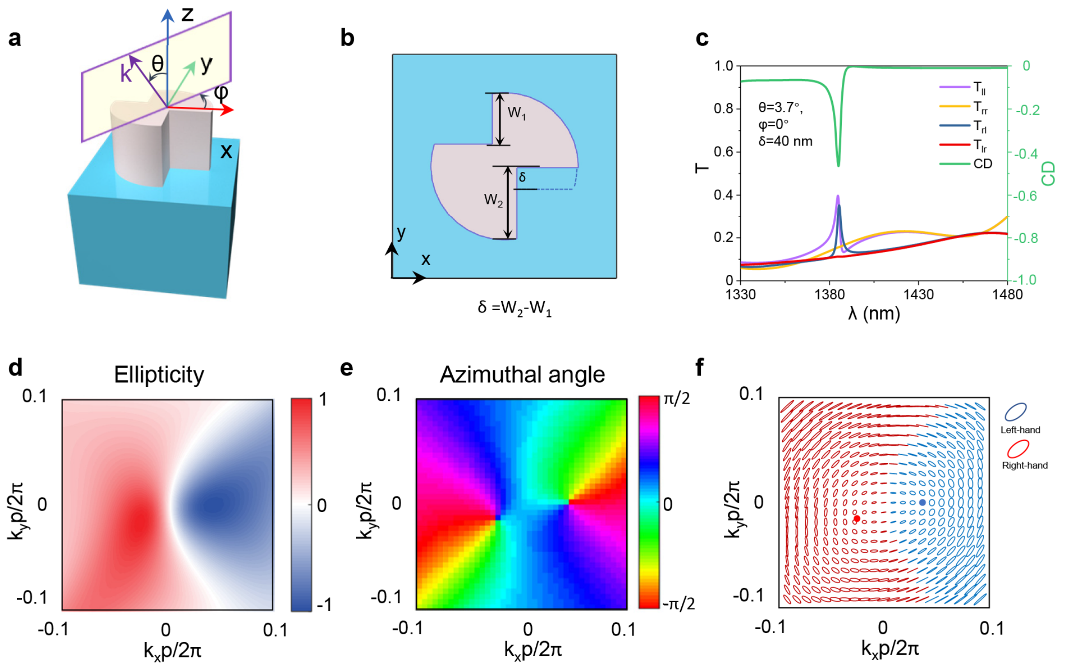

Figure S4. Planar chiral q-BIC metasurface with $\delta \neq 0$ supporting circular eigen-polarization (*C* points). (a, b) Schematic of the metasurface (δ=40 nm) with the parameters: P=850 nm, H=350 nm, R=280 nm, L_1=220 nm, W_1=191 nm, L_2=230 nm, and W_2=231 nm illuminated with incident angle θ and conical angle φ. (c) Jones matrix elements and the CD spectra under the incidence parameters of θ=3.7°, φ=0°, which correspond to the blue C point in (f). (d, e) Color maps of the eigen-polarization states in the k-space (d: ellipticity χ, e: azimuthal angle ψ). (f) Eigen-polarization ellipse distribution in the k-space. The blue and red represent the eigen left-handed states and right-handed states.

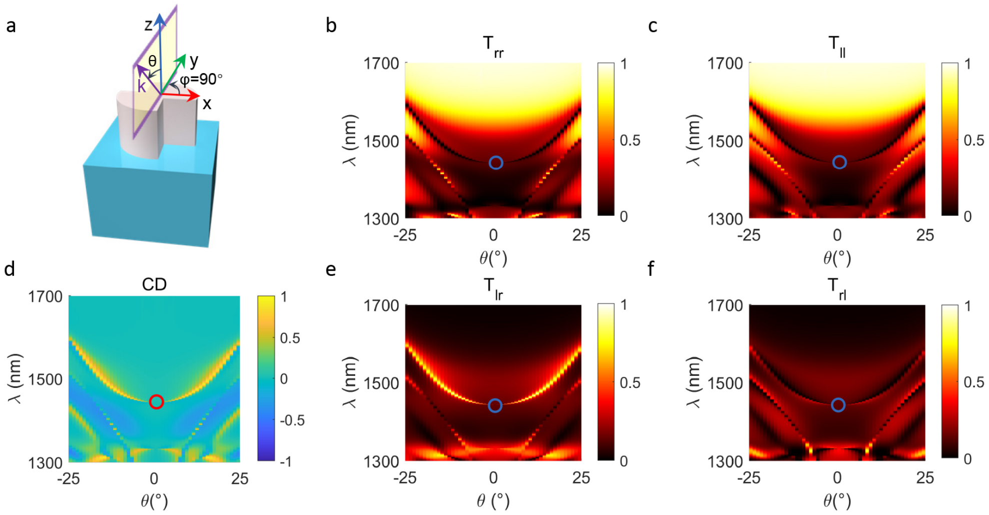

Figure S5. Evolutions of transmission spectra of all Jones matrix elements and CD spectra at oblique incidences along the φ=90° direction. (a) Schematic of the q-BIC metasurface (δ=0) with parameters P=850 nm, R=280 nm, L_1=L_2=220 nm, W_1=W_2=191 nm, and H=350 nm for varying incident angles θ along the φ=90° direction. The transmission spectra (b, c, e, f) of all Jones matrix elements (T_{lr}, T_{rl}, T_{rr}, T_{ll}) and CD spectra (d) of the planar chiral q-BIC metasurface.

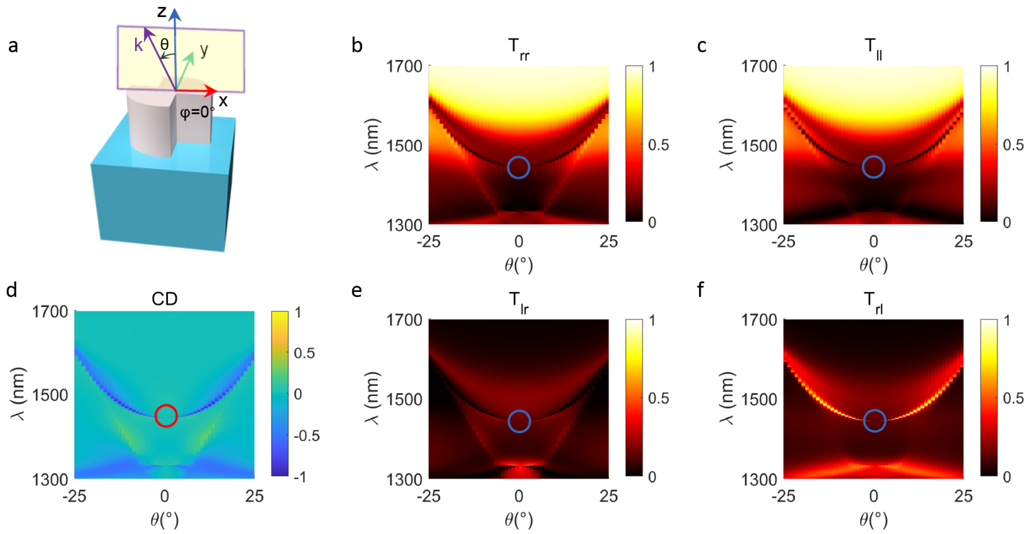

Figure S6. Evolutions of transmission spectra of all Jones matrix elements and CD spectra at oblique incidences along the φ=0° direction. (a) Schematic of the q-BIC metasurface with the same parameters as Fig. S5 (δ=0) for varying incident angles θ along the φ=0° direction. The transmission spectra (b, c, e, f) of all Jones matrix elements (T_{lr}, T_{rl}, T_{rr}, T_{ll}) and CD spectra (d) of the planar chiral q-BIC metasurface.

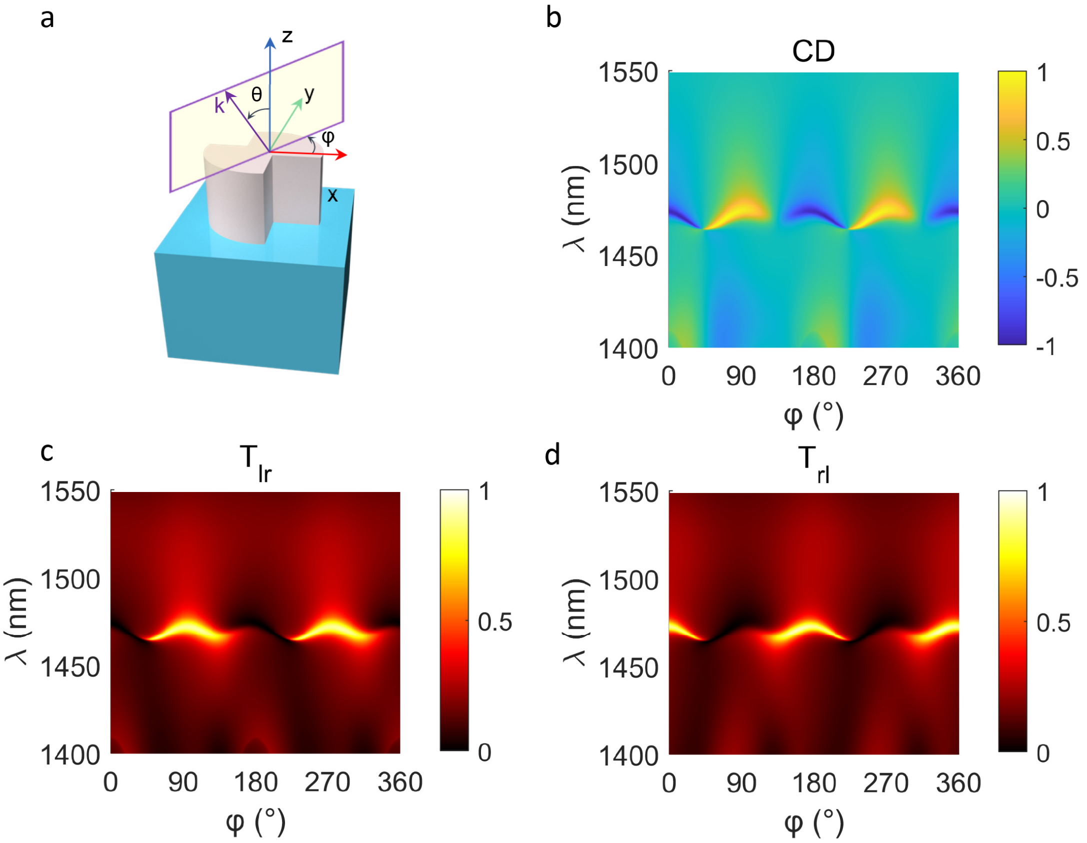

Figure S7. CD and transmission spectra evolution along the conical angle direction. (a) schematic of the q-BIC metasurface with δ=0 at incident angle θ=12° along the φ-direction. (b-d) The CD spectra and the transmission efficiency spectra of T_{lr} and T_{rl} with respect to φ and wavelength. When the incidence light evolves a circle along conical directions (φ evolves from 0° to 360°), the opposite varying trend of the RCP and LCP transmission can be observed. With the increase of φ, the sign of CD flips at the positions near [(2n-1)×45°, (n=1,2,3,4)].

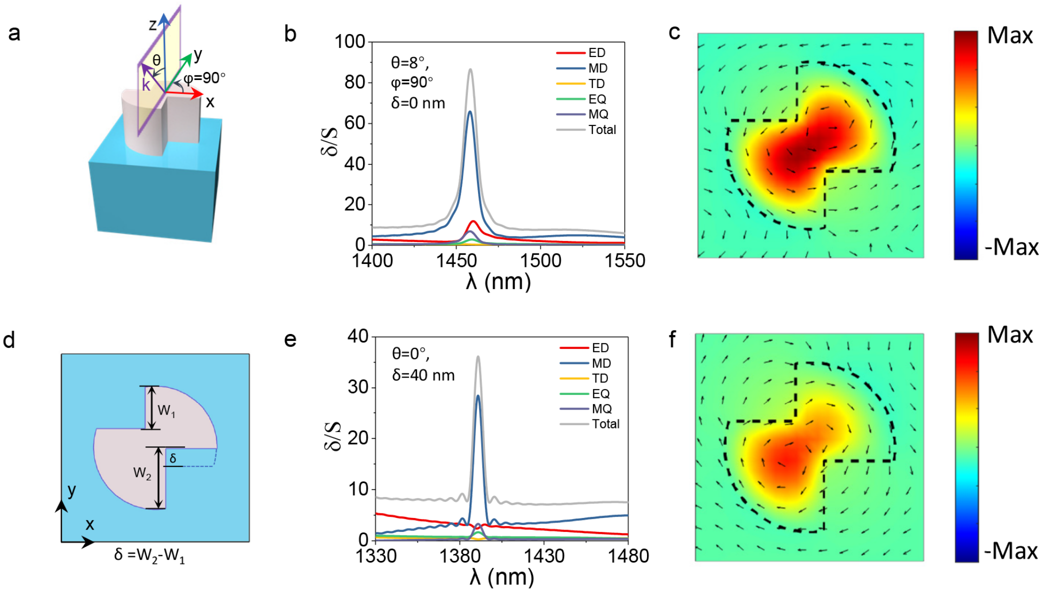

Figure S8. Multipole expansions and nearfield electromagnetic patterns of the planar chiral q-BIC metasurface. (a, d) Schematic of the symmetry-breaking processes that transfer the BIC to planar chiral q-BICs. (b, e) Scattering cross-section spectra by multipole expansions of the planar chiral q-BIC metasurface (b) with the same parameters as Fig. S5 (δ=0) for RCP incidences at θ=8°, φ = 90° and (e) with the same parameters as Fig. S4 (δ=40 nm) for RCP normal incidences. (c, f) The magnetic field H_z (color) and electric field E_x, E_y (vector) of the MD mode, corresponding to the planar chiral q-BIC mode at (c) λ=1456 nm, and (f) at λ=1392 nm, respectively.

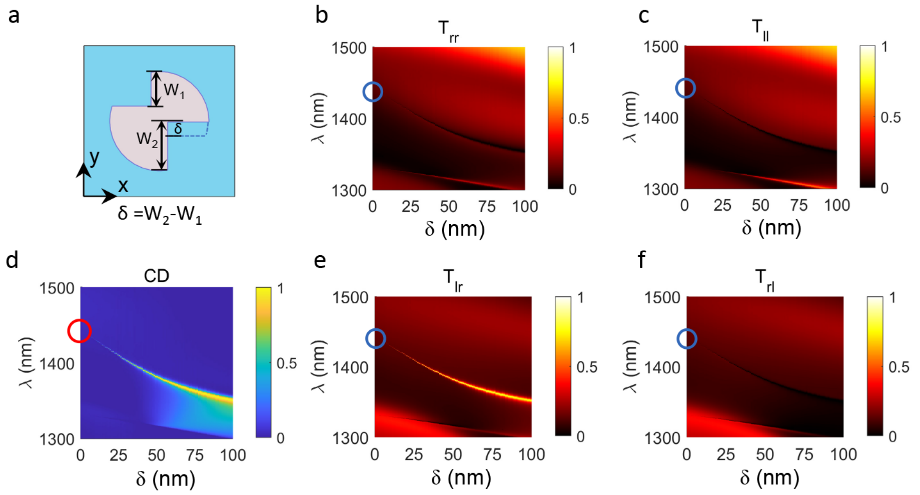

Figure S9. Evolutions of transmission spectra of all Jones matrix elements and CD spectra with varying parameter *δ*. (a) Schematic of the q-BIC metasurface with varying parameter *δ*. The transmission spectra (b, c, e, f) of all Jones matrix elements (T_{lr}, T_{rl}, T_{rr}, T_{ll}) and CD spectra (d) of the planar chiral q-BIC metasurface.

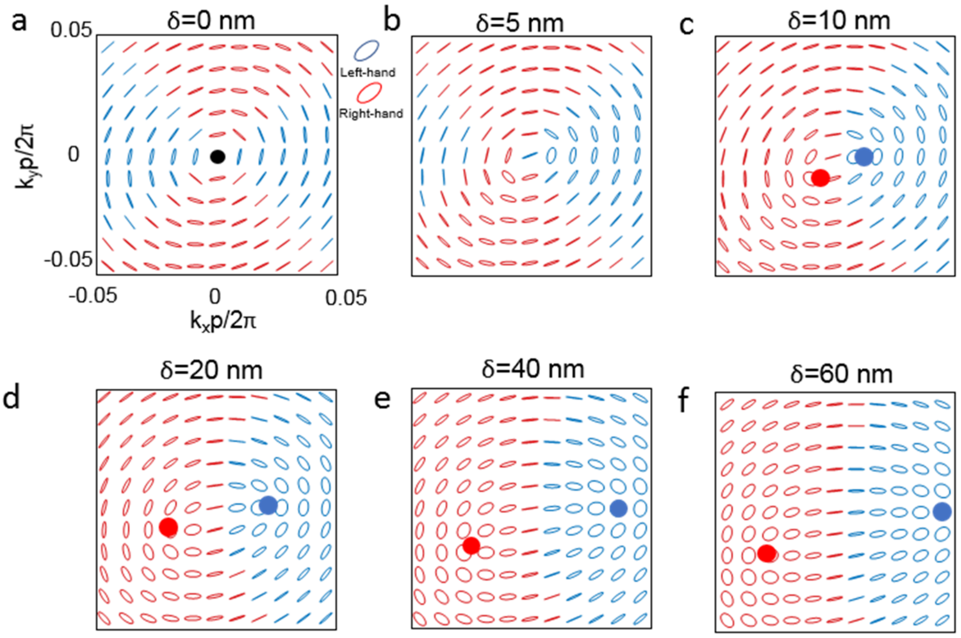

Figure S10. Eigen-polarization ellipse distribution in the *k*-space with different *δ*. The blue and red represent the eigen left-handed states and right-handed states. The red/blue dot represents the circular eigen-polarization state, and the black dot represents the *V* point (BIC).

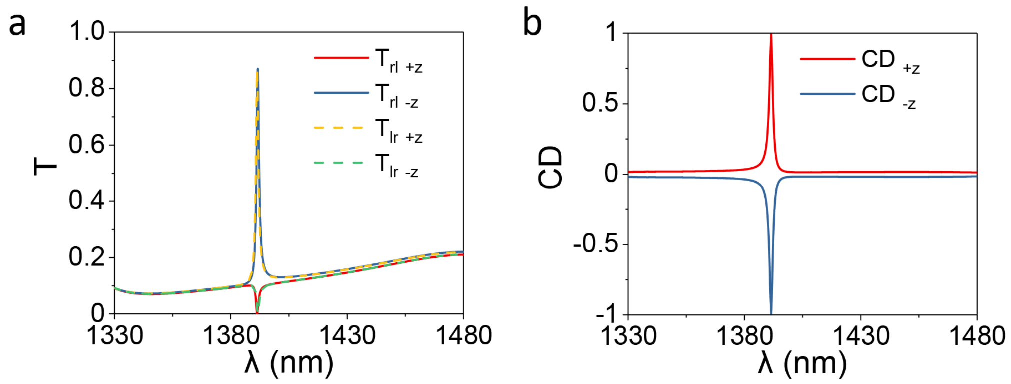

Figure S11. Intrinsic planar chirality with asymmetry transmission. Transmission efficiency spectra of (a) T_{rl}, T_{lr} and (b) CD in the propagation direction from -z to +z (represented by subscript +z in the figure) and in the propagation direction from +z to -z (represented by subscript -z in the figure) for the in-plane symmetry-breaking metasurface (δ=40 nm). The spectral lines of T_{lr}, and T_{rl} are opposite with the reversed CD when the incident direction is reversed. This is due to the mirror symmetry in the out-of-plane direction. The planar optical chirality is represented by the circular polarization conversion between the output light and the incident light. When the propagation direction is reversed, the chirality is reversed, which is the asymmetric transmission phenomenon accompanied by the planar chirality.

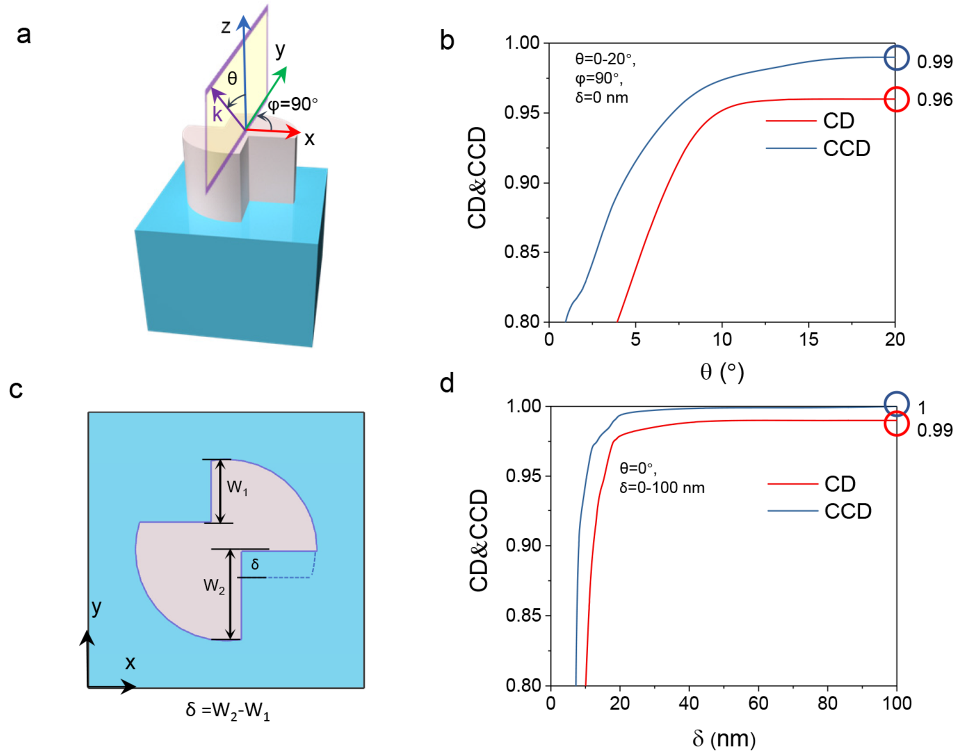

Figure S12. Comparison of CD and CCD spectra at the q-BIC peak. Here, CCD is defined as the difference between polarization conversion components: $CCD = \frac{T_{lr}-T_{rl}}{T_{lr}+T_{rl}}$. (a, c) Schematic of the symmetry-breaking processes that transfer the BIC to planar chiral q-BICs. (b, d) The evolution of CD and CCD spectra by (b) continuous varying incident angle θ along the φ=90° direction with δ=0 and by (d) the in-plane geometry asymmetry parameter δ.

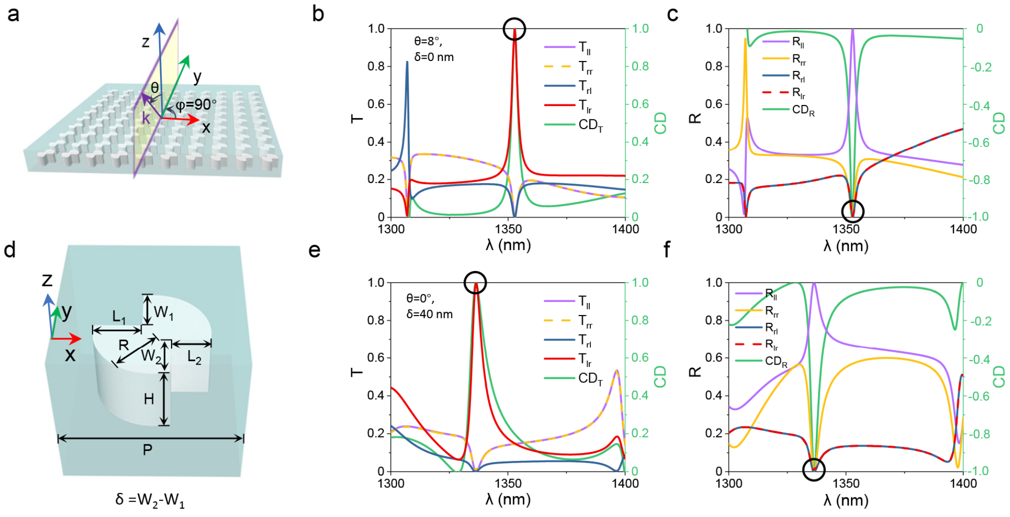

Figure S13. Perfect unitary circular conversion transmittance T_{lr} and CD can be achieved by strictly symmetric background. (a, d) Schematic of the symmetry-breaking processes that transfer the BIC to planar chiral q-BICs when the metasurface is placed in a completely symmetric background (n_{air}=1). All Jones matrix elements of the simulated (b, e) transmission and (c, f) reflection as well as the CD spectra with (b, c) parameters: P=850 nm, R=300 nm, L_1=L_2=119 nm, W_1=W_2=175 nm, and H=450 nm (δ=0) under both LCP and RCP incidences at θ=8°, φ=90°, and (e, f) with parameters P=850 nm, R=300 nm, L_1=310 nm, W_1=209 nm, L_2=319 nm, W_2=249 nm, and H=575 nm (δ=40 nm). The CD_T can ideally reach 1 with T_{ll}=0, T_{rr}=0, T_{rl}=0 and T_{lr}=1 at the working wavelength. The reflection spectra R_{lr} are ideally equal to R_{rl}, while R_{ll} has a q-BIC peak and R_{rr} has a dip, which is opposite to the case for transmission spectra. The CD_R ideally reaches -1 with R_{ll}=1, R_{rr}=0, R_{rl}=0 and R_{lr}=0 at the working wavelength.

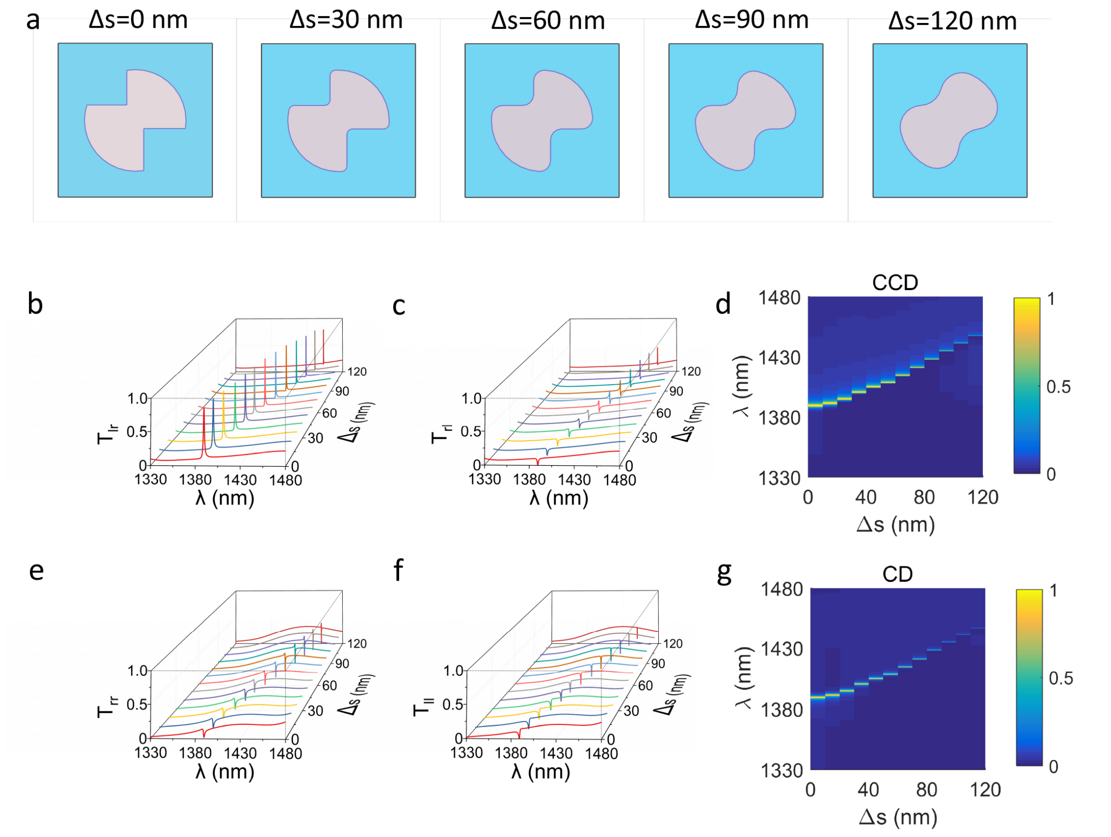

Figure S14. Structure tolerance for fabrication. (a) Schematic of the planar q-BIC chiral metasurfaces with different manufacturing tolerance parameter △s. Five representative unit cells with different △s are shown on the top panel, where △s=0 nm, 30 nm, 60 nm, 90 nm, and 120 nm. (b, c, e, f) Jones matrix spectra (T_{lr}, T_{rl}, T_{rr} and T_{ll}) of the metasurfaces for different △s. (d, g) CCD and CD of the metasurfaces for different △s. As we can see, the structure always maintains superb planar chiral q-BIC properties when the structure chamfer radius increases in a large range (from 0 to 120 nm), implying that our design has a relaxed fabrication precision requirement. The results of Fig. 3 (illumination symmetry breaking) show similar resonance widths, while for Fig. 4 (broken meta-atom in-plane inversion symmetry) the experiment is broader than simulation. This discrepancy is related to the structure tolerance of the fabricated structure. The structure in Fig. 3 (δ=0) is the easiest one for experimental fabrication, because this structure is completely symmetrical and the radian of each side is relatively large. Under other parameters, especially for δ=80 nm, the radian at the long edge is very small and the corner angle is very sharp. The fabrication precision demand for those parameters is more stringent, leading to the discrepancy of the bandwidth between the simulation and experimental results. Generally speaking, the larger the $\boldsymbol{\delta}$, the more difficult for precise fabrication and the less consistency between the simulation and experimental results.

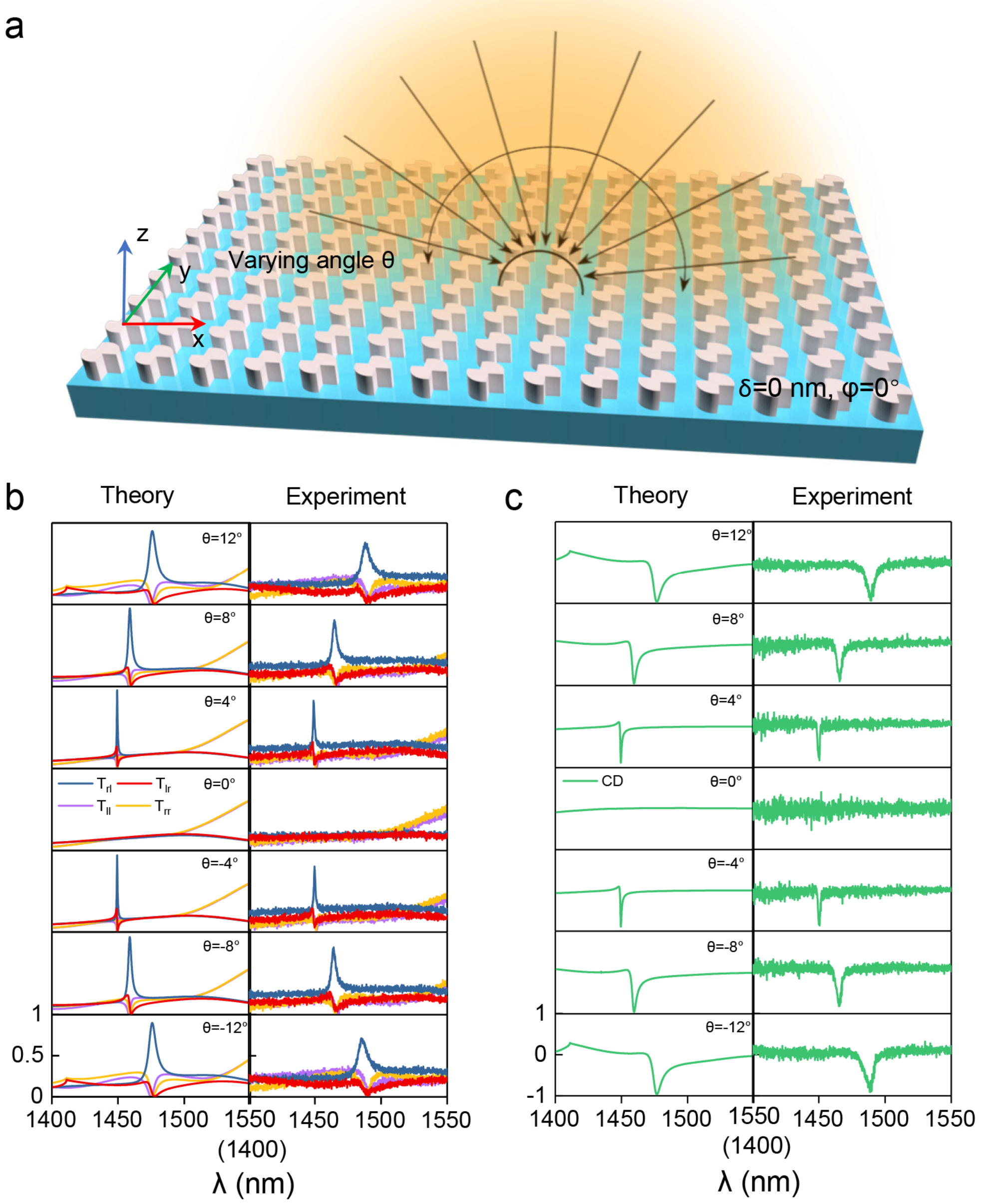

Figure S15. Experimental verification of the extrinsic planar chiral q-BIC with illumination symmetry breaking. (a) Schematic of the δ=0 metasurface supporting chiral q-BICs at different incident angles along the φ=0°direction. (b) Simulated and measured transmission Jones matrix spectra (T_{lr}, T_{rl}, T_{rr} and T_{ll}) for different incident angles (θ=0°, ±4°, ±8°, ±12°) along the φ=0° direction. (c) Simulated and measured CD spectra for different incident angles extracted from the Jones matrix spectra in (b).

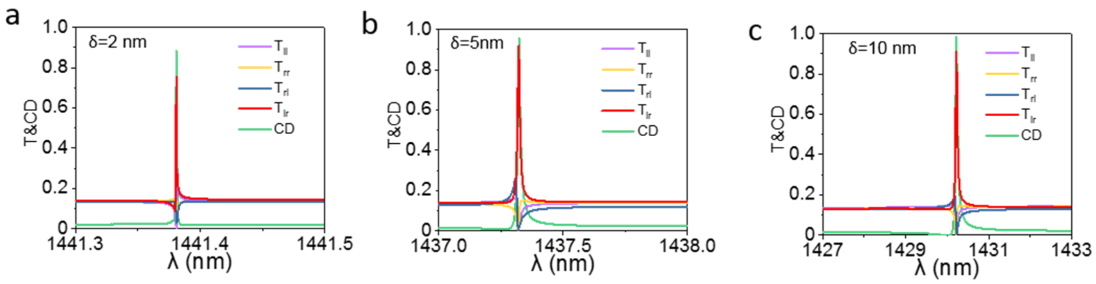

Figure S16. Simulated transmission Jones matrix spectra of T_{ll}, T_{rr}, T_{rl} and T_{lr} as well as the CD spectrum of the metasurface with extremely small asymmetry parameter δ=2 nm, 5 nm, and 10 nm for normal incidences. The simulated Q-factor and corresponding CD reach 800416/0.88, 66527/0.95, 12768/0.98, respectively.

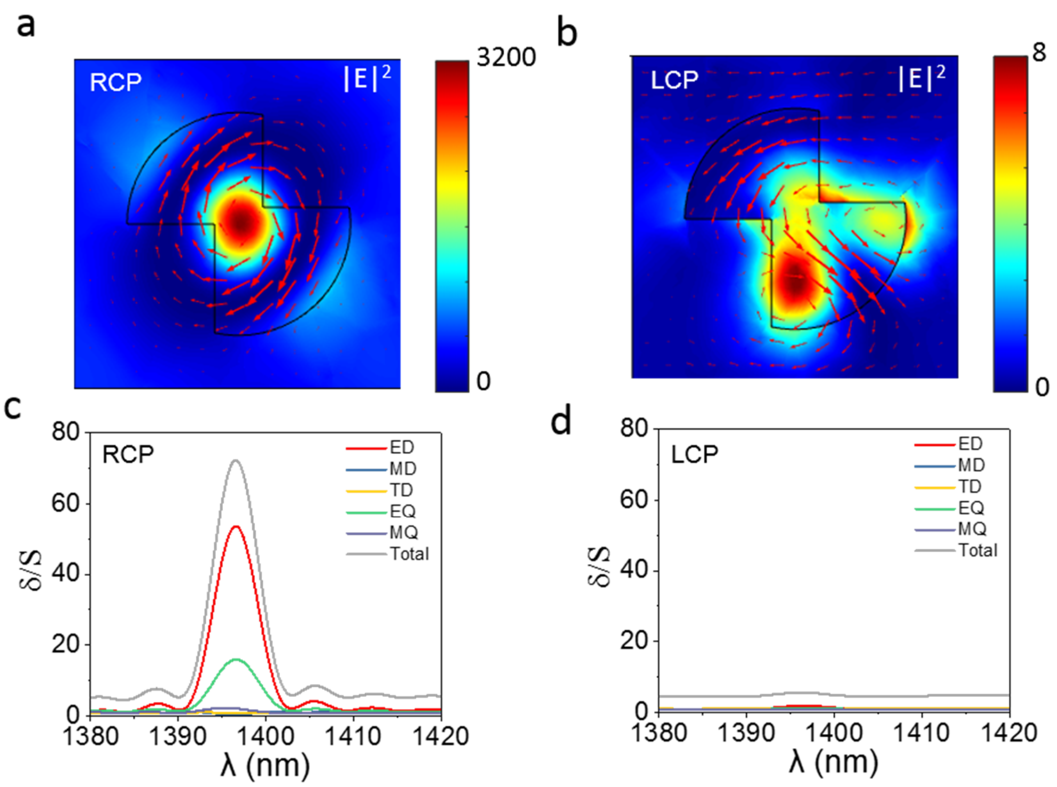

Figure S17. Nearfield electromagnetic patterns and multipole expansions of the planar chiral q-BIC metasurface with circular eigen-polarization (parameters are the same as that of Fig. 5 in the maintext). (a, b) The electric field $|E|^2$ (color) and magnetic field H_x, H_y (vector) for (a) RCP and (b) LCP normal incidences at λ=1396 nm. (c, d) Scattering cross-section spectra by multipole expansions of the planar chiral q-BIC metasurface with δ=40 nm for (c) RCP and (d) LCP normal incidences.

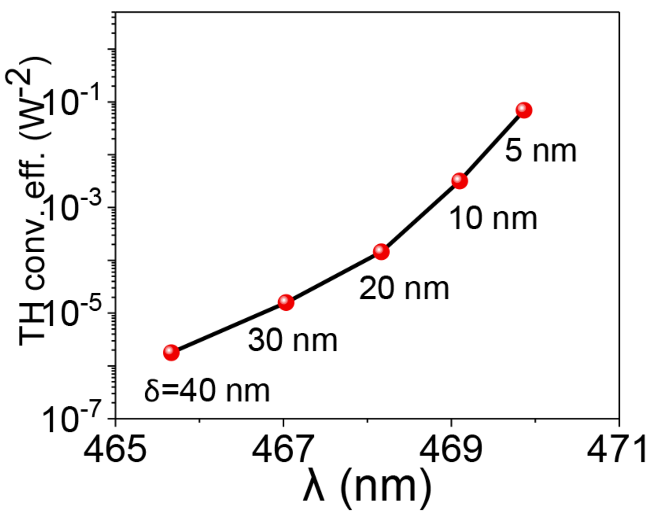

Figure S18. ultrahigh TH conversion efficiency with the planar chiral q-BIC metasurface with circular eigen-polarization (parameters are the same as that of Fig. 5 in the maintext). Simulated TH conversion efficiency of the metasurface with δ =5 nm, 10 nm, 20 nm, 40 nm for RCP incidences.

References

1. Fan, S., Suh, W. & Joannopoulos, J. D. Temporal coupled-mode theory for the Fano resonance in optical resonators. *J. Opt. Soc. Am. A* **20**, 569-572 (2003).
2. Suh, W., Wang, Z. & Fan, S. Temporal coupled-mode theory and the presence of non-orthogonal modes in lossless multimode cavities. *IEEE J. Quantum Electron.* **40**, 1511-1518 (2004).
3. Zhou, H., *et al.* Perfect single-sided radiation and absorption without mirrors. *Optica* **3**, 1079-1086 (2016).
4. Liu, W., *et al.* Circularly polarized states spawning from bound states in the continuum. *Phys. Rev. Lett.* **123**, 116104 (2019).
